# Assessing the feasibility of applying machine learning to diagnosing non-effusive feline infectious peritonitis

**DOI:** 10.1038/s41598-024-52577-4

**Published:** 2024-01-30

**Authors:** Dawn Dunbar, Simon A. Babayan, Sarah Krumrie, Hayley Haining, Margaret J. Hosie, William Weir

**Affiliations:** 1 School of Biodiversity, One Health and Veterinary Medicine, College of Medical, Veterinary and Life Sciences, University of Glasgow, Glasgow, G61 1QH UK; 2 MRC-University of Glasgow Centre for Virus Research, University of Glasgow, Glasgow, G61 1QH UK

**Keywords:** Diagnostic markers, Infectious diseases, Computational models, Data mining, Machine learning

## Abstract

Feline infectious peritonitis (FIP) is a severe feline coronavirus-associated syndrome in cats, which is invariably fatal without anti-viral treatment. In the majority of non-effusive FIP cases encountered in practice, confirmatory diagnostic testing is not undertaken and reliance is given to the interpretation of valuable, but essentially non-specific, clinical signs and laboratory markers. We hypothesised that it may be feasible to develop a machine learning (ML) approach which may be applied to the analysis of clinical data to aid in the diagnosis of disease. A dataset encompassing 1939 suspected FIP cases was scored for clinical suspicion of FIP on the basis of history, signalment, clinical signs and laboratory results, using published guidelines, comprising 683 FIP (35.2%), and 1256 non-FIP (64.8%) cases. This dataset was used to train, validate and evaluate two diagnostic machine learning ensemble models. These models, which analysed signalment and laboratory data alone, allowed the accurate discrimination of FIP and non-FIP cases in line with expert opinion. To evaluate whether these models may have value as a diagnostic tool, they were applied to a collection of 80 cases for which the FIP status had been confirmed (FIP: n = 58 (72.5%), non–FIP: n = 22 (27.5%)). Both ensemble models detected FIP with an accuracy of 97.5%, an area under the curve (AUC) of 0.969, sensitivity of 95.45% and specificity of 98.28%. This work demonstrates that, in principle, ML can be usefully applied to the diagnosis of non-effusive FIP. Further work is required before ML may be deployed in the laboratory as a diagnostic tool, such as training models on datasets of confirmed cases and accounting for inter-laboratory variation. Nevertheless, these results illustrate the potential benefit of applying ML to standardising and accelerating the interpretation of clinical pathology data, thereby improving the diagnostic utility of existing laboratory tests.

## Introduction

Feline infectious peritonitis (FIP) is a viral disease of cats caused by feline coronavirus (FCoV). FIP is described pathologically as a severe immune-mediated vasculitis or perivasculitis that affects multiple organs and results in a disease from which cats do not naturally recover^1,2^. The prevalence of FCoV infection in the feline population is high and FCoV is considered ubiquitous in environments where cats exist. In the UK, approximately 26% of cats are seropositive^3^ and in multi-cat environments, such as shelters, this increases to between 50 and 100%^4^. FCoV primarily causes self-limiting, mild or asymptomatic enteric infection in cats and only in a minority of cases, around 5%, does FIP develop. Until recently, FIP was invariably fatal as no effective treatment was available to control the virus or the pathology it induces. Fortunately, effective therapeutics are now available and so swift and accurate diagnosis can improve animal survival and welfare, allowing treatment to be instigated at an early stage^5,6^. While evidence of FCoV infection in feline patients is easily attained through both serological testing and direct pathogen detection^7^, the utility of such tests to diagnose FIP is limited when they are used in isolation. In addition, the clinical presentation of FIP overlaps extensively with several other feline diseases^8^ making it a major diagnostic challenge.

FIP comprises a spectrum of clinical presentations, including ‘effusive’ and ‘non-effusive’ disease^9,10^, the former being characterised by the presence of an abdominal or pleural effusion^11^. Currently, the only accepted method of confirming a diagnosis of non-effusive FIP is histopathological examination of diseased organs, ideally accompanied by anti-FCoV immunohistochemistry (IHC), although this is usually only possible post-mortem^12,13^. In practice, only a small proportion of cases are confirmed ante-mortem using IHC, in part because this involves invasive sampling of sick cats, and in part because no effective treatment options were previously available in the event of a confirmed diagnosis. For this reason, a myriad of ante-mortem tests have been developed over the decades, but each has its limitations^10,14^.

For the majority of cases, the de facto methodology for diagnosing FIP relies on the use of a complex decision-making system requiring a panoply of laboratory test results together with expert clinical interpretation. The Advisory Board for Cat Diseases (ABCD) has published a series of recommended diagnostic approaches for FIP, which illustrates the complexities surrounding the interpretation of clinical signs and the diagnostic tests utilised^15^. On the basis of history, signalment, clinical signs and laboratory tests, a high level of suspicion of FIP can be determined. However, these guidelines are complex and rely on the clinician to integrate evidence from dozens of clinical parameters, each of which can at best only provide semi-quantitative evidence of infection. For example, fever is classed as likely to be present, while icterus is moderately likely to be present and pallor is only slightly likely to be present. Equally, a history of fighting or being feral makes it slightly and moderately more unlikely for the cat to have FIP, respectively. The variable nature of FIP presentation, which is influenced by the stage of progression, means that a single, authoritative decision-making tree cannot be constructed, making FIP diagnosis particularly challenging for the primary care clinician.

The concept of using machine learning (ML) as a diagnostic tool has existed for several decades^15–19^. We hypothesised that using ML algorithms to interpret blood chemistry, haematology and serology data could aid clinicians in the ante-mortem diagnosis of FIP. ML has recently been employed for the diagnosis of COVID-19^20,21^ and has also been used in the risk assessment, diagnosis and prognosis of diseases including Alzheimer’s^22^, diabetes comorbidities^23^, cancer^24,25^ and various infectious diseases^26,27^. More recently, ML has been employed within veterinary medicine in areas such as diagnostic image analysis^28,29^, prediction of chronic kidney disease^30^, the early detection of leptospirosis^31^ and predicting the risk of bovine viral diarrhoea outbreaks^32^. Indeed Pfannschmidt et al*.* used FIP as a case study to illustrate the application of some principles of ML to disease diagnosis^33^.

Here we utilise an extensive, curated database of veterinary clinical data and clinical pathology laboratory results and assess their utility to create accurate and reliable informatic-based models for diagnostic classification of suspect FIP cases. To determine whether, in principle, an ML model could be developed as a diagnostic tool, we elected to utilise a dataset consisting of nearly two thousand cases which has been interpreted in accordance with accepted published clinical guidelines^15^. Using this methodology, cases are not defined in terms of particular threshold values or the definitive presence or absence of specific markers. They are instead defined on the balance of evidence, classifying cases on the basis of history, clinical signs, signalment and laboratory measures. As these classifications were, by definition, reliant on subjective expert opinion, it was necessary to evaluate model performance on a set of eighty cases on which a definitive diagnosis had been reached. The reliance on expert opinion versus a gold standard for model training is a limitation of this study, a further limitation is the use of markers both for manual classification and model training; both are discussed herein. Despite these limitations, we present this work as proof-of-principle that ML has value in the analysis of FIP laboratory data and we report the development and performance of such models.

## Methods

### Dataset and data preparation

Cases submitted to the Veterinary Diagnostic Services (VDS) laboratory between 2001 and 2021 with a suspicion of non-effusive FIP were considered for enrolment in this study. A set of laboratory parameters and case metadata provided by submitting clinicians was abstracted from the VDS Laboratory Information Management System (LIMS). The laboratory data included the following variables measured on blood: anti-FCoV antibody titre, alpha-1-glycoprotein (AGP), total protein, albumin, globulin, albumin:globulin ratio, haemoglobin, red blood cell count, haematocrit, mean corpuscular volume, mean cell haemoglobin, mean cell haemoglobin concentration, total white cell count, band neutrophils, neutrophils, lymphocytes, monocytes, eosinophils, basophils and normoblasts. Demographic data including age, sex and pedigree and clinical notes, denoted as “reason” on the LIMS, was also collected. Retrospective diagnostic disease classifications, based on expert clinical interpretations, were also collected from the LIMS system alongside the laboratory data. A minimum of three clinicians were involved in the decision-making process, including at least one clinical pathologist, and classifications were based on consensus opinion. The personnel providing the interpretations differed across the years. These interpretations were used as ‘ground truth’ for classifying whether samples represented FIP cases or not in the training, validation and expert opinion test datasets.

Cases in which expert clinical opinion (based on the signalment, clinical history and laboratory results), following the ABCD FIP diagnostic guidelines^15^ current at the time of interpretation, indicated an extremely high suspicion of non-effusive FIP were included in the analysis, as were cases where non-effusive FIP was not considered a differential diagnosis. The cases included in the study were ill cats with a wide range of clinical presentations, where FIP was considered a differential diagnosis by the referring clinician, and the FIP profile was performed as part of a diagnostic workup. Cases were designated as “high suspicion of FIP” where sufficient criteria within the ABCD guidelines were met to warrant this classification, based on a combination of signalment, history, clinical signs and laboratory data. Similarly, a set of cases was identified where there was a strong suspicion that the cat did not have FIP. The creation of strict case definitions based on a defined set of clinical and laboratory features was avoided as this is not achievable in line with the balanced interpretation of ABCD guidelines^15^ and, importantly, it prevented the creation of artefactual models which are overfitted to subsets of parameters within the case defining criteria. Cases in which the interpretation was equivocal were excluded; these included cases where FIP was still considered a differential, but there was insufficient evidence to strongly support or refute an FIP diagnosis. Similarly, cases with incomplete records, due to only a subset of tests being performed, were excluded as some ML algorithms are unable to cope with missing data. This may have been due to insufficient material being submitted or the incorrect sample type being provided. Only data from cases representing initial FIP diagnostic submissions were included; re-test and follow-up test data was excluded, as were suspect cases where treatment with anti-virals, namely GS-441524, Remdesivir or equivalents, had already commenced. Cases where treatment had commenced with palliative drugs, such as steroidal or non-steroidal anti-inflammatories, or with antibiotics were not excluded from the study.

The dataset, comprising cases with expert clinical interpretation, was randomly partitioned into three smaller datasets for modelling purposes namely “training”, “validation” and the “expert opinion test set”, which comprised 40%, 40% and 20% of the total records, respectively. An additional set of 80 reference cases with histology and/or IHC and/or PCR or an alternative non-FIP diagnosis were used as a “gold standard” dataset to evaluate the effectiveness of the models. A summary of the signalment and laboratory variables included in the model development and evaluation datasets is detailed in Table 1.

**Table 1:** Description of variables used in the training, validation, expert opinion test datasets and definitive diagnosis cases.

Variable (unit)	Method	Mean/median (*) or number (range or %)						Reference interval
		Expert opinion (for training & validation)		Expert opinion (for evaluation)		Gold standard (for evaluation)		
		Non-FIP group (n = 1013)	FIP group (n = 538)	Non-FIP group (n = 243)	FIP group (n = 145)	Non-FIP group (n = 58)	FIP group (n = 22)	
Serology								
Anti-FCoV antibodies	Immunofluorescent antibody test (IFAT)	0* (0–1920)	1920* (640–1920)	0* (0–1920)	1920* (640–1920)	0* (0–1920)	1920* (1280–1920)	
Haematology								
Haemoglobin (g/dL)	Siemens Advia 120 haematology system	10.75 (1.4–22)	7.805 (2.2–14.2)	10.62 (3.70–17.2)	7.614 (3.3–12.9)	10.4 02 (2.63–16.1)	7.905 (4.40–14.5)	10–15.0
Neutrophils (× 10^9^ cells/L)		9.072 (0.016–73.6)	11.615 (0.510–47.301)	9.309 (0.043–49.1)	12.117 (0.761–39.565)	8.296 (1.571–33.524)	12.301 (2.598–27.747	2.5–12.5
Lymphocytes (× 10^9^ cells/L)		2.354 (0.055–22.099)	1.284 (0–6.883)	2.171 (0.038–20.299)	1.315 (0–6.092)	3.158 (0.122–50.828)	1.944 (0–6.013)	1.5–7
Monocytes (× 10^9^ cells/L)		0.404 (0–6.16)	0.388 (0–3.413)	0.412 (0–2.66)	0.425 (0–2.64)	0.385 (0–2.576)	0.3796 (0.041–1.088)	0–0.85
Eosinophils (× 10^9^ cells/L)		0.404 (0–8.235)	0.12 (0–2.391)	0.413 (0–6.591)	0.11 (0–1.977)	0.458 (0–5.643)	0.182 (0–1.201)	0–1.5
Biochemistry								
α-1-acid glycoprotein (mg/mL)	RID/ELISA	1213 (300–3601)	2394 (340–3601)	1242 (300–3601)	2356 (780–3601)	1087 (300–3380)	2529 (720–3601)	0–500
Albumin (g/L)	Siemens dimension Xpand Plus (biochemistry analyser)	29.72 (12–45)	21.99 (13–36)	29.53 (15.0–46.0)	22.6 (9.0–39.0)	29.28 (17.0–37.0)	23.5 (18–35)	26-36
A:G ratio		0.712 (0.16–3.21)	0.307 (0.13–0.7)	0.709 (0.1–1.57)	0.309 (0.17–0.63)	0.693 (0.29–1.12)	0.371 (0.19–0.9)	
Demographic								
Age (years)		4* (0–20)	1* (0–14)	4* (0–16)	1* (0–13)	3* (0–13)	0* (0–9)	
Sex		n = 579 (57.2%) male, n = 434 (42.8%) female	n = 366 (68%) male, n = 172 (32%) female	n = 147 (60.5%) male, n = 96 (39.5%) female	n = 93 (64.1%) male, n = 52 (35.9%) female	n = 31 (53.5%) male, n = 27 (46.5%) female	n = 15 (68.1%) male, n = 7 (31.9%) female	
Pedigree		n = 248 (24.5%) pedigree, n = 765 (75.5%) non-pedigree	n = 255 (47.4%) pedigree, n = 283 (52.6%) non-pedigree	n = 66 (27.2%) pedigree, n = 177 (72.8%) non-pedigree	n = 72 (49.7%) pedigree, n = 73 (50.3%) non-pedigree	n = 20 (34.5%) pedigree, n = 38 (65.5%) non-pedigree	n = 16 (72.7%) pedigree, n = 6 (27.3%) non-pedigree	

Datasets are stratified by classification group (non-FIP or FIP). The method of testing, mean or median, range of variables and the reference interval as appropriate for variables are detailed for n = 1939 expert opinion cases, n = 80 definitive diagnosis cases. *IFAT* immunofluorescent assay, *RID* radial immunodiffusion, *ELISA* enzyme linked immunosorbent assay.

### Feature selection

Statistical analysis of all variables, comprising correlation and covariance, was undertaken to establish redundancy across each variable within the dataset. In addition, expert guidance was sought from clinical pathologists regarding potentially redundant variables from a clinical perspective. Together, this allowed the number of features used in the modelling exercise to be reduced. Model-based feature importance was also examined throughout the modelling process, during both the training and validation stages. Features were assessed by model-based feature importance using the base learner models: Logistic Regression (LR), Naïve Bayes (NB), Support Vector Machine (SVM), randomForest (rF) and Extreme Gradient Boosting (XGBoost). Where the models allowed, Gini index was assessed for feature importance. For the Naïve Bayes and SVM models, these model types are not compatible with calculating Gini index, therefore model accuracy was instead assessed using iterative removal of features.

### Model selection and building

A range of algorithms was selected to incorporate into the models, each exhibiting a different underlying mathematical or statistical methodology. This approach sought to test our hypothesis in a comprehensive manner, without bias towards any particular algorithm. Five types of classification algorithm were implemented, namely Logistic Regression, Naïve Bayes, Support Vector Machine, randomForest and Extreme Gradient Boosting.

Binary classification models were trained using predictor variables listed in Table 1 and these were either numerical variables or dummy binary variables (numeric type) coded “0” or “1” (‘one-hot encoding’) for a specific group. The response variables for the models were also coded as binary variables (numeric type), “0” for cases classified as not-FIP and “1” for cases classified as FIP. All variables, both predictor and response, were coded as numeric data as some algorithms required a numerical matrix as the input data.

**Figure 1:**
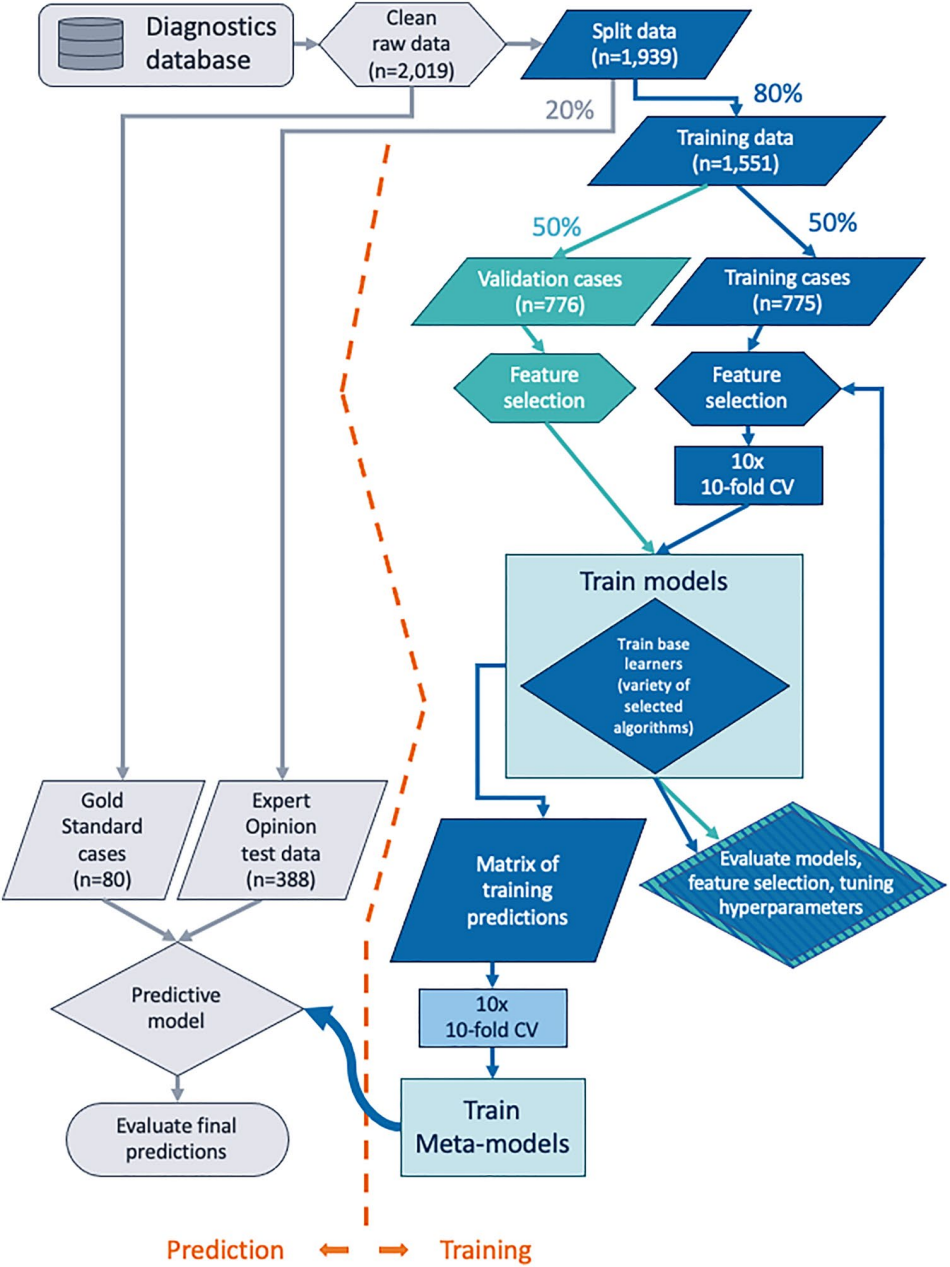
Data handling, processing and model building steps. *CV* cross-validation.

Models were built in the statistical programming language *R* (version 4.1.2)^34^. The caret package (version 6.0–85)^35^ was used to access the data pre-processing and algorithm functions. Figure 1 illustrates the workflow from data collection through the process of model building and evaluation to final predictions.

Two predictive binary classification ensemble models were built employing the algorithms listed previously. The training dataset used to train all models, base learners and the logistic regression models was pre-processed using "Caret” pre-processing functions. Data was centred and scaled, and the “downSample” function was used to randomly generate an input dataset where the frequency of both outcome classes was the same as the minority class (FIP cases in this instance). Data used as input for validation and evaluation were similarly centred and scaled through the model function, however there was no requirement to “down-sample” these datasets.

The first approach, the XGBoost ensemble, used one hundred XGBoost base learner models, the predictions of which were then aggregated into an input array for a final stacked randomForest predictive model. The second mixed ensemble comprised one hundred base learner models in total, consisting of 25 randomForest, 25 Naïve Bayes, 25 logistic regression and 25 SVM models. As with XGBoost ensemble, the output predictions of the base learners were aggregated into an array and used as input for a final randomForest ensemble model. The function “caretStack” (from caretEnsemble) was used to build each ensemble random forest model (meta-model) and used ten-fold cross-validation, repeated ten times; the number of randomly drawn candidate variables at each split was forced to 40 (the “mtry” hyperparameter of the random forest algorithm) to ensure that a representative selection of the base learner predictions was evaluated.

Each base learner was trained using ten-fold cross-validation, repeated ten times. The “caretList” function (from caretEnsemble) was used to build the base learners and a grid of optimal tuning parameters was selected for each base learner model using the validation dataset. The model building function automatically selected the best tuning parameters from the parameter grid and consequently selected the optimal model at each iteration; selection was based on accuracy and kappa of the cross-validation hold-out data. Tuning parameters varied for each base learner. Optimised tuning grids are provided in Supplementary Table S1. An additional mixed ensemble model was built as above but without the use of FCoV titre or AGP as predictive variables; all other parameters remained the same.

### Basic logistic regression models

We built two standalone basic logistic regression models as comparators to the more complex ensemble models. The logistic regression models, similar to those included in our ensemble models, did not have any hyperparameter tuning performed. One was trained with the same set of predictors as the ensembles and the other with FCoV serology as a single predictor (with ten-fold cross-validation used in each).

### Model evaluation and statistical analysis

Ten-fold cross-validation was undertaken with nine folds being used for training and the remaining fold used to evaluate accuracy during training. Additional evaluation of model performance was undertaken using three subsequent datasets. The validation data were used to fine tune the base learners and evaluate tuning parameters, and therefore these data were not used to evaluate the final models to avoid information leakage. The final testing was performed using the “expert opinion” test dataset (a partition of expert interpreted cases) and a group of reference cases (“gold standard”) where either pathology, histopathology, IHC, PCR or a combination thereof was used to determine a definitive diagnosis of FIP, or an alternative diagnosis was determined.

Models were assessed as though they were a new diagnostic tool; confusion matrices were generated using the model predictions and the expert predicted outcome was used as the reference. Model performance metrics including accuracy, sensitivity, specificity and inter-rater agreement (Cohens Kappa, Κ) were used to compare each model’s predictions with the actual outcome. The area under the receiver operator curve (AUC) was also calculated. The basic logistic regression models were also evaluated in this way. A corrected McNemar test^36,37^ was used to assess statistical significance of differences between sensitivity and specificity of different models.

All statistical analyses were performed using the statistical programming language “*R”* (version 4.1.2)^34^ and the packages “Base R”, “stats” (version 4.1.2) and “pROC” (version 1.18.0)^38^. “Tidyverse” (version 1.3.1) was used for data preparation, cleaning and graphical output of results. “Caret (version 6.0.90)”^35^ and “caretEnsemble” (version 2.0.1) were used to build models, to predict from trained models and to produce confusion matrices. “Caret” is a wrapper for several other packages and has several package dependencies from which the algorithms themselves originate; the dependencies utilised for the algorithms are listed in Supplementary Table S1. Additional packages used throughout are listed in Supplementary Fig. S1. An alpha level (*P*-value) < 0.05 was considered statistically significant in all analyses. The R Studio IDE (version 2021.09.1) was used to facilitate the use of the R language.

### Ethical approval

This study was authorised by the School of Veterinary Medicine Ethics Committee, University of Glasgow, application number EA46/21.

## Results

### Case summary

**Figure 2:**
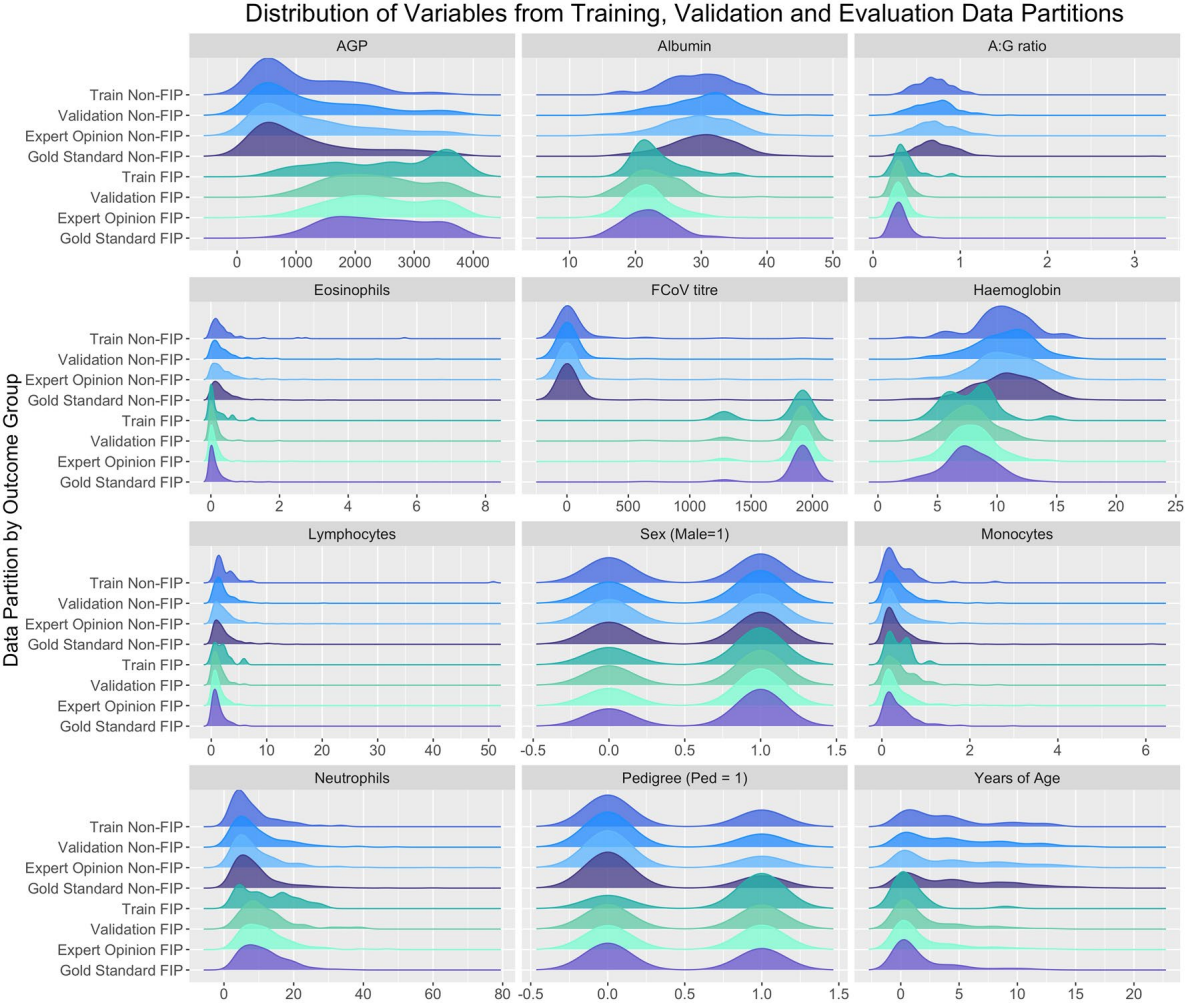
Density plots of the variables included in the models. Expert opinion cases (greens and blues) stratified by outcome classification and data partition and gold standard cases (purples) stratified by outcome classification.

A total of 2019 eligible cases was used in this study, representing cases where case history, clinical signs, signalment, laboratory data and clinical interpretation was all available. A collection of 1939 (96%) cases where clinical interpretation was used as a proxy for “ground truth” was used in the training and development stages; in 683 (35%) of cases there was strong evidence to support a diagnosis of FIP while for the remaining 1256 (65%) of cases, the evidence did not support a diagnosis of FIP. A total of 775 (40%) cases was randomly “down-sampled” during pre-processing in each base learner model build to produce a balanced dataset with which to train the models, with an even split of positive and negative cases. This was distinct from the validation dataset which consisted of 776 (40%) cases with a bias towards non-FIP cases n = 510 (65.7%), and an expert opinion testing dataset n = 388 (20%) which contained a similar proportion of non-FIP cases n = 243 (62.6%). A set of 80 gold standard cases, where either FIP had been confirmed or an alternate diagnosis was reached, was used for final evaluation. A summary of the variables used in the analysis, stratified by dataset and disease classification group, is displayed in Table 1. This is accompanied by Fig. 2 which shows the distributions of the data points, again stratified by dataset and classification group. A summary of the cat population analysed is provided in Table 2.

**Table 2:** Summary of population characteristics for all cases.

Characteristic			No of patients in cohort (%)			
			Interpreted cases (n = 1939)		Confirmed cases (n-80)	
			Non-FIP group (n = 1256)	FIP group (n = 683)	Non-FIP group (n = 58)	FIP group (n = 22)
Sex	Male	Entire	237 (18.9)	211 (30.9)	12 (20.7)	6 (27.3)
		Neutered	489 (38.9)	248 (36.3)	19 (32.8)	9 (40.9)
	Female	Entire	218 (17.4)	129 (18.9)	8 (13.8)	5 (22.7)
		Neutered	312 (24.8)	95 (13.9)	19 (32.8)	2 (9.1)
Age (years)	0 to 1		295 (23.5)	329 (48.2)	1 (17.2)	14 (63.6)
	1 to 2		249 (19.8)	184 (26.9)	17 (29.3)	7 (31.8)
	3 to 5		260 (20.7)	96 (14.1)	15 (25.9)	–
	5 to 10		304 (24.2)	59 (8.6)	11 (19.0)	1 (4.5)
	10 to 20		148 (11.8)	15 (2.2)	5 (8.6)	–
Breed	Not specified		28 (2.2)	16 (2.3)	–	–
	DSH/DLH/X		913 (72.7)	340 (49.8)	38 (65.5)	6 (27.3)
	Pedigree		315 (25.1)	327 (47.9)	20 (34.5)	16 (72.7)
			Siamese (49), Bengal (41), British Shorthair (40), Maine Coon (32), Persian Longhair (26), Birman (23), Ragdoll (19), Burmese (14), Oriental (8), Norwegian Forest Cat (8), British Blue (6), Other pedigree n ≤ 5 (49)	Birman (62), British Shorthair (51), Ragdoll (38), Bengal (36), Maine Coon (22), Siamese (16), Burmese (16), Persian Longhair (13), British Blue (12), Oriental (8), Devon Rex (6), Norwegian Forest Cat (6), Other pedigree n ≤ 5 (41)	Bengal (5), Siamese (4), Oriental (2), Persian Longhair (1), Maine Coon (1), Ragdoll (1), Cornish Rex (1), British Blue (1), Norwegian Forest Cat (1), Burmese (1), Siberian (1), Egyptian (1)	Bengal (4), Ragdoll (3), Burmese (2), Persian Longhair (1), Maine Coon (1), Devon Rex (1), Russian Blue (1), British Shorthair (1), Siamese Seal Point (1), Birman (1)

### Exploratory analysis

**Figure 3:**
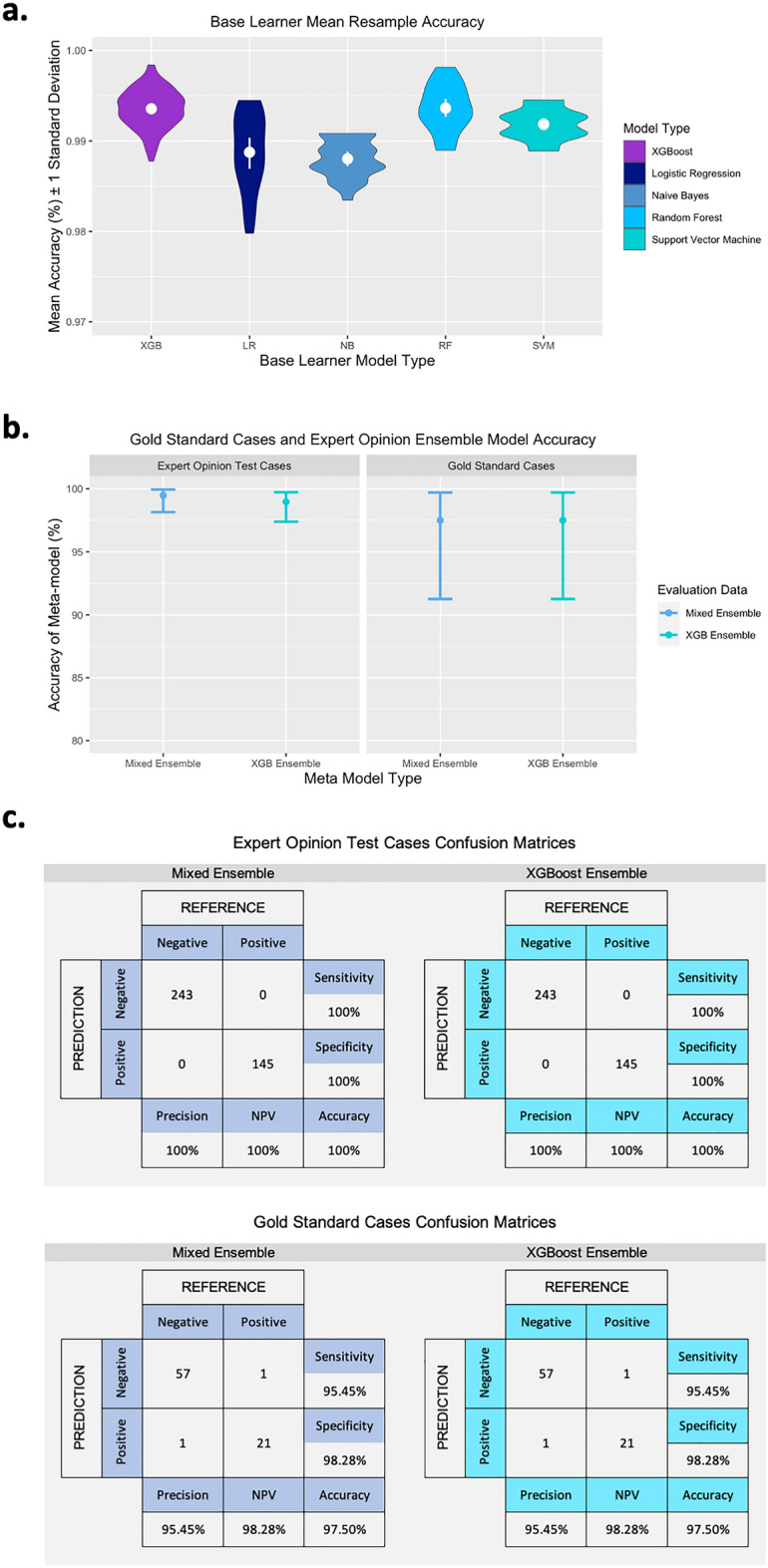
Principal component analysis using all available variables. The labelled vectors illustrate the direction and force of effect (length of the vector) that the original variables have on PC1 and PC2.

The clinical laboratory dataset was initially subject to principal component analysis; the first two principal components (PC) are illustrated in Fig. 3 and together PC1 and PC2 explain 45% of the variation in the dataset. Cases with high suspicion of non-effusive FIP clustered together in one group and non-FIP cases clustered in a second group. As anticipated, these clusters showed some degree of overlap.

Distributions of datapoints across outcome classification groups (i.e. ‘FIP’ and ‘Non-FIP’) and data partitions are shown in Fig. 2, illustrating that each outcome classification is associated with a representative dataset.

### Feature importance

**Figure 4:**
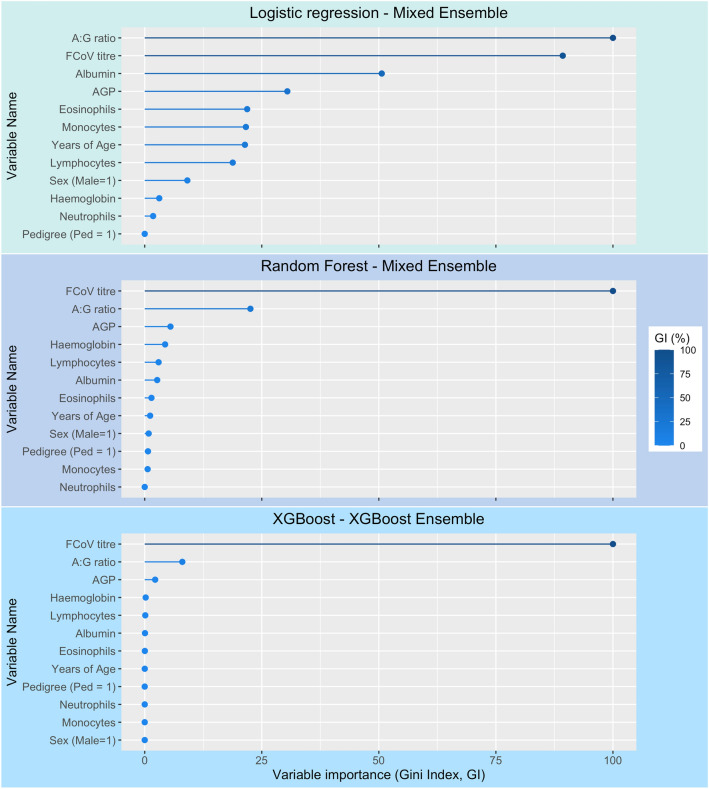
Selected examples of variable importance as assessed by Gini index within algorithms and model builds. These illustrate the difference in variable importance between algorithms and within the ensemble models. *GI* Gini index.

All variables are listed above in the data preparation section. These were reduced down to the model variables by means of feature selection, which is described as follows. Highly correlated variables and those that showed covariance were removed from the dataset. Correlation of remaining variables is illustrated in Supplementary Fig. S2. The initial data contained three measures that could each have been treated as a proxy for anaemia; only haemoglobin measurement was retained, as it was least likely to be affected by artefactual changes during transit to the laboratory. Some white blood cell (WBC) counts (i.e. band neutrophils, basophils) and normoblasts were removed as there were insufficient cases with levels above zero or with values recorded. Haematology experts agreed that these were not informative variables for FIP diagnosis. The overall WBC count was removed as the absolute counts for neutrophils, lymphocytes, monocytes and eosinophils were considered more informative. Albumin and A:G ratio were retained as they were deemed more informative, while total protein and globulins were removed as the latter are derivatives of these measures, and correlate highly with albumin and A:G ratio. “Reason” contains the clinical history or notes provided by a submitting clinician; this was not utilised in this modelling exercise as the data was not encoded in a state that was readily usable. Supplementary Table S2 shows a full list of the variables removed through the feature selection process and the rationale in each case. During evaluation of model-based feature importance, we observed that variable importance varied greatly between base learner models. All features that showed importance to any model were retained so as not to disadvantage any other models. A selection of variable importance plots is shown in Fig. 4; these show the differences in variable importance between base learners. No further variables were removed during model-based feature selection.

### Model performance

#### XGBoost ensemble model

##### Base learners

**Figure 5:**
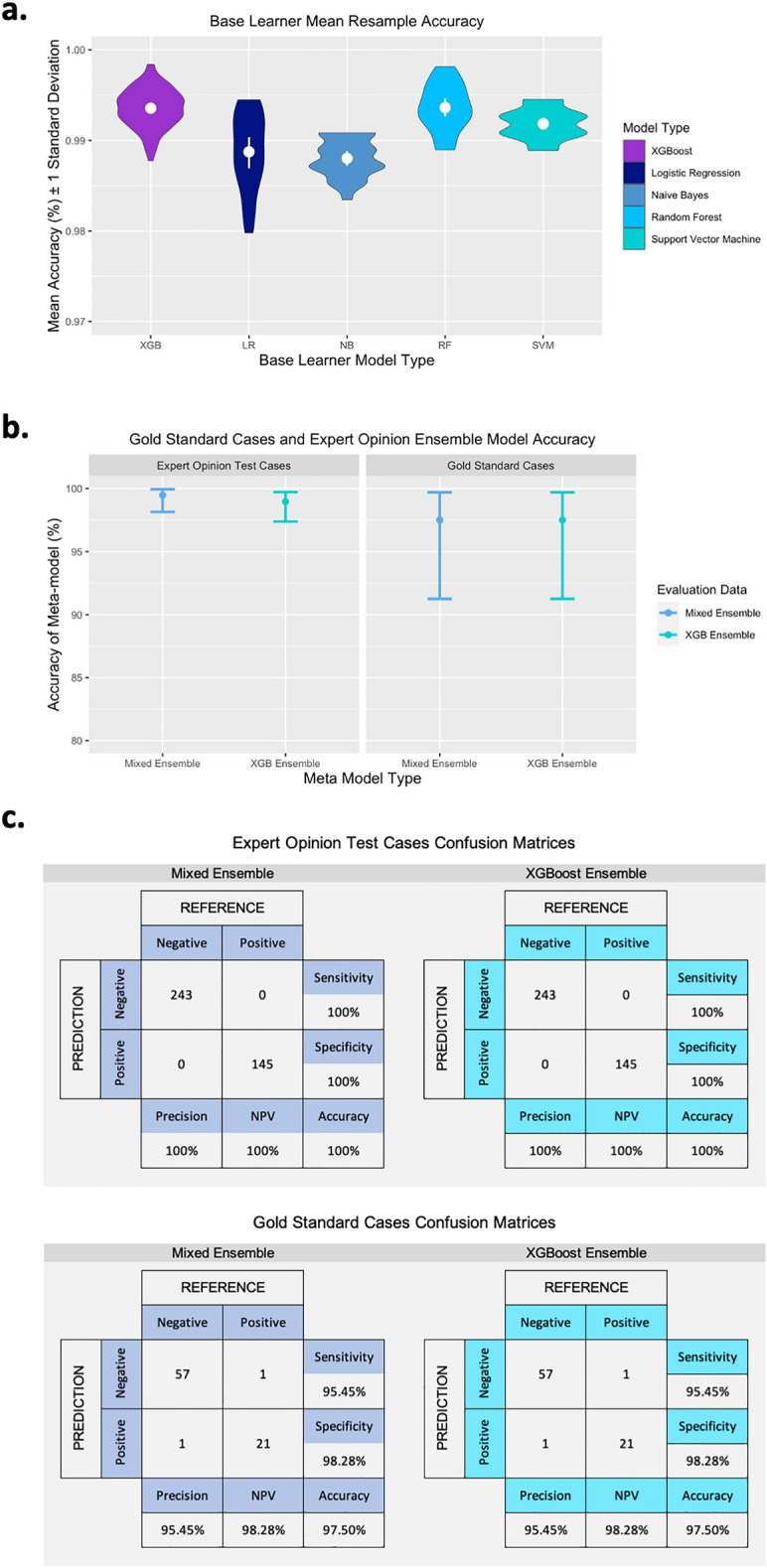
Accuracy measures of all models. (**A**) Mean resample accuracy of the base learner models from both ensemble models, XGB n = 100, n = 25 for the other base learners. (**B**) Model accuracy assessed using gold standard case data and expert opinion test data with 95% CI. (**C**) Confusion matrices for the gold standard case data and expert opinion test data. *NPV* negative predictive value.

The base learner XGBoost models showed an in-model mean resample accuracy of 99.35% (range 98.77–99.84%) as measured on the cross-validation hold-out fold (Fig. 5A). The validation data was used to tune the base models and select optimal tuning parameters (see Supplementary Table S1 for final model tuning parameters).

##### Meta-models

A mean training resample accuracy of 100% was achieved on the random forest ensemble model. The model accuracy on the validation data was 99.36% (95% CI 98.5–99.79%). Overall accuracy measures are illustrated in Fig. 5B and additional measures are presented in Table 3. Using the expert opinion test data, the models had an accuracy of 100.0% (95% CI 99.05–100.0%) as measured by a confusion matrix (Fig. 5C) which comprised model predictions and pre-classified outcome, and an AUC of 1 (95% CI 1.0–1.0). When applied to the gold standard cases, the ensemble models demonstrated an accuracy of 97.5% (95% CI 91.26–99.7%) and an AUC of 0.969 (95% CI 0.921–1.0), with a single false positive and a single false negative prediction (Fig. 5C).

**Table 3:** Model accuracies for each ensemble model with each dataset.

Evaluation dataset	Model	Base learner training resample accuracy (%) (range)	Validation accuracy (%) (95% CI)	Accuracy (%) (95% CI)	Sensitivity (%) (95% CI)	Specificity (%) (95% CI)	Cohens kappa (κ)	AUC (95% CI)
Training & validation	XGBoost ensemble	99.35 (98.77–99.84)	99.36 (98.5- 99.79)*	–	99.62 (98–100)	99.22 (98–100)	98.57	0.994 (0.989–1.0)
	Mixed ensemble	99.05 (97.97–99.81)	99.48 (98.69–99.86)*	–	99.62 (98–100)	99.41 (98–100)	98.86	0.995 (0.990–1.0)
Expert opinion test set	XGBoost ensemble	–	–	100 (99.05–100)*	100 (98–100)	100 (97–100)	100	1.0 (1.0–1.0)
	Mixed ensemble	–	–	100 (99.05–100)*	100 (98–100)	100 (97–100)	100	1.0 (1.0–1.0)
Gold standard cases	XGBoost ensemble	–	–	97.5 (91.26–99.7)*	95.45 (77–100)	98.28 (91–100)	93.73	0.969 (0.921–1.0)
	Mixed ensemble	–	–	97.5 (91.26–99.7)*	95.45 (77–100)	98.28 (91–100)	93.73	0.969 (0.921–1.0)

Accuracy, sensitivity, specificity, kappa and AUC of each model are detailed.

*Both ensembles had a P-value < 0.05 for the accuracy measure on the confirmed cases data. This P-value is for the significance of the accuracy compared to the no information rate (NIR), i.e. the model predicting the outcomes by chance.

#### Mixed ensemble model

##### Base learners

The base learners for the mixed ensemble models were individual models built using logistic regression, support vector machine, random forest and Naïve Bayes algorithms. The models showed an in-model resample accuracy of 99.05% (range 97.97–99.81%) as measured on the cross-validation hold-out fold overall. The individual model accuracy breakdowns are shown in Fig. 5A. The validation data was used to evaluate the final base models and associated tuning parameters (see Supplementary Table S1 for parameter lists).

##### Meta-models

Again, a mean training resample accuracy of 100% was achieved on the random forest ensemble model. The validation data provided an accuracy level of 99.48% (95% CI 98.69–99.86%). Overall accuracy measures are illustrated in Fig. 5b and additional measures are presented in Table 3. When applied to the expert opinion dataset the models showed an accuracy of 100.0% (95% CI 99.05–100.0%) as measured by a confusion matrix (Fig. 5c) and an AUC of 1 (95% CI 1.0–1.0). The gold standard cases, when assessed using the mixed ensemble model, had an accuracy of 97.5% (95% CI 91.26–99.7%) and an AUC of 0.969 (95% CI 0.921–1.0), again illustrated in (Fig. 5c) where there was one false positive and one false negative prediction. Both ensemble models were compared across evaluation datasets using the McNemar test and there was no statistical difference in the sensitivity and specificity between compared models (Supplementary Table S3).

#### Mixed ensemble model without FCoV or AGP

##### Base learners

The base learners for the mixed ensemble models were individual models built using logistic regression, support vector machine, random forest and Naïve Bayes algorithms. The models showed an in-model resample accuracy of 99.06% (range 97.98–99.81%) as measured on the cross-validation hold-out fold overall. The validation data was used to evaluate the final base models and associated tuning parameters (see Supplementary Table S1 for parameter lists).

##### Meta-models

A mean training resample accuracy of 92.02% (range 90.07–94.12%) was achieved on the random forest ensemble model. The validation data provided an accuracy level of 92.91% (95% CI 90.87–94.62%). Supplementary Table S4 displays all results for this model. The expert opinion test data produced an accuracy of 92.27% (95% CI 89.15–94.72%) as measured by a confusion matrix and an AUC of 0.931 (95% CI 0.907–0.956). The gold standard cases, when assessed using the mixed ensemble model minus FCoV and AGP, had an accuracy of 92.5% (95% CI 84.39–97.2%) and an AUC of 0.920 (95% CI 0.850–0.990), and there were four false positive and two false negative predictions. When compared to the mixed ensemble and the XGBoost ensemble with all predictors, there was no statistical difference in the sensitivity and specificity when applied to the gold standard data. However, the ‘no FCoV, no AGP’ model was significantly less accurate than both ‘all variable’ ensembles (p-value < 0.05 on McNemar test) when assessed on both the validation and the expert opinion datasets (Supplementary Table S3).

#### Basic logistic regression models

The logistic regression model was the most basic base learner. With no tuning parameters, this model with the full set of predictors had an accuracy of 95% (95% CI 87.69–98.62%) and an AUC of 0.951 (95% CI 0.898–1.0); the model misclassified four out of 80 cases. The model with FCoV as a single predictor had an accuracy of 97.5% (95% CI 91.26–99.7%) and an AUC of 0.983 (95% CI 0.959–1.0), misclassifying two out of 80 cases. The full results of the basic logistic regression models are reported in Supplementary Table S4.

Both LR models were compared to both ensemble models using the McNemar test and there was no statistical difference in the sensitivity and specificity (Supplementary Table S3).

### Minimum panel of variables

Whilst evaluating the data for modelling, we established a minimum panel of reliable variables which may be used for standard FIP diagnostic inference. These variables include measurements and signalment data which are highly informative, least likely to be affected by artefactual changes and which do not correlate or co-vary with other measures. These variables are listed in Table 1.

## Discussion

This study evaluated whether ML models could be trained on a retrospective set of cases where FIP status could be inferred from the interpretation of history, clinical signs, signalment and laboratory data, in line with consensus guidelines^15^. Our findings revealed that ML has the potential to accurately predict disease status in agreement with expert clinical opinion. Based on signalment and laboratory data alone, unsupervised exploratory analysis suggested there were nuanced patterns within this dataset, which would enable discrimination between samples from FIP and non-FIP groups. The two-dimensional PCA shown in Fig. 3 illustrates the first two PCs, which demonstrate that FIP and non-FIP cases fall into two discernible clusters. Subsequently, we were able to develop highly accurate predictive models using a variety of methods. The accuracy and generalisability of these models are promising; values for both sensitivity and specificity greater than 95% on new and unseen data constitute excellent test performance. While these results are favourable and illustrate the feasibility and potential for using machine learning in FIP diagnosis, it is important to appreciate the limitations of the present study and the need for further work before a deployable model is ready. The accuracy metrics from classifying test datasets based on clinical opinion are very likely over-estimations of the true ‘real world’ accuracies of the models. Firstly, despite there being no data leakage through the data processing and model building process, the predictive models use a subset of the data used during clinical case classification, and so an element of over-fitting of the models is likely. Secondly, the ‘ground truth’ for the current models is based on expert clinical opinion using consensus diagnostic guidelines^15^, rather than a gold standard case dataset. Therefore, any systematic biases in clinician’s opinion resulting in incorrectly inferring the true FIP status of a case could be carried through to the models. For this reason, should they become available, datasets with hundreds of cases diagnosed by gold standard could be used to refine these models. Encouragingly, with the advent of effective anti-viral therapeutics, FIP is considered a treatable condition, and for this reason one might predict that the proportion of suspected cases having confirmatory testing undertaken will only rise. While this raises hope that an effective ML tool could eventually be developed based on the diagnostic test results from a single laboratory, to develop a generalised method that can be applied to results from different laboratories would remain a major challenge. This would involve either standardising methodologies to a common analytical framework or developing models that could take account of data from different haematology/biochemistry analysers and serological methods. The most likely scenario is, perhaps, that a range of laboratory-specific models is created that are trained on datasets generated on equipment in-house. Despite further work being required before a deployable ML model is created, it is encouraging to note the excellent performance of the models on the eighty gold standard cases in the present study; their accuracy of 97.5% and AUC of 0.969 demonstrates they have genuine potential as a predictive tool.

In addition to developing preliminary predictive models, we have identified a minimum panel of measures and factors that may form the basis of future models (Table 1). The vectors illustrated on the PCA reinforce the utility of these features to discriminate between disease classification groups and the high level of performance of the models on the ‘gold standard’ dataset is testament to their diagnostic potential. We also looked at feature importance at various stages of model building; Fig. 4 illustrates the feature importance of a selection of base learner models, generating further evidence that the features included in our modelling are useful in disease classification. Unfortunately, these values are not easily computed for Support Vector Machine or Naïve Bayes models, therefore early in the developmental stages we iteratively removed these features and assessed the impact on the predictions. Ultimately, due to the diversity of the algorithms employed and the range of features used across the model iterations, we opted to retain all the features listed in Table 1, as they contributed to at least some of the models. The initial data contained three measures that could each have been treated as a proxy for anaemia; only haemoglobin measurement was retained, as it was least likely to be affected by artefactual changes during transit to the laboratory. We thoroughly examined the other measures prior to exclusion, and there was found to be no detriment to disease prediction upon their removal. Our analysis indicated that high FCoV titres and high levels of AGP combined with low A:G ratio and reduced haemoglobin levels were major markers associated with FIP, in line with the ABCD guidelines^15^. FCoV infection is a prerequisite to FIP, AGP is a non-specific marker for inflammation, A:G ratio indicates hyperglobulinaemia and lower haemoglobin corresponds with anaemia, and all are consistently recognised as being important but non-specific clinical markers of FIP. Also significant, albeit to a lesser extent, was the aberration in white blood cell composition, which contributed informatively to the discrimination of FIP from non-FIP disease. Signalment factors (age, sex and pedigree) were also important, but to a lesser extent than the haematological and biochemical changes.

In addition to the ensemble models, we evaluated two basic logistic regression models. These both displayed a high degree of accuracy, and it could be argued that these possessed some potential for development as predictive models for FIP diagnosis. It could be inferred from these LR models that FCoV titre alone is a valuable biomarker with respect to FIP disease-status prediction when used to assess a cohort of cats such as the one used for this study. The LR models built on FCoV titre alone generated results comparable to our ML models illustrating that in this cohort of FIP suspected cats FCoV titre has a high positive predictive value (PPV) and is a strong predictor for FIP. However, from clinical studies, it is already well-recognised in the veterinary diagnostics field that single markers, such as FCoV seropositivity, alone should not be used as a predictor for FIP^15^. Though this marker performs well in this specific cohort, reliance upon it would not generalise to query cases where diagnosis is more complex and where major disease markers such as antibody titre and AGP level are equivocal. Consequently, we decided to investigate whether the other haematological and biochemical markers alone possessed a discernible diagnostic signal by proceeding to build mixed ensemble models excluding these two markers. Encouragingly, the predictive capacity of these ensemble models remained high, demonstrating the detection of nuanced informative patterns in the haematology and biochemistry results alone.

ML is inherently flexible to variations in the data, where more traditional statistical models such as logistic regression, are static and far less flexible. To overcome this limitation, a focus of this study was building ensemble models, which represent a range of different base algorithms. In addition to the future work suggested above, benefit will be gained by incorporating confirmed cases into the training set where the initial clinical suspicion of disease is lower, and where systemic markers may not be as markedly perturbed, such as in cases of neurological or uveitis-associated FIP. The individual base learners might not perform as well in predicting outcomes in isolation for less ‘extreme’ cases, however it is possible that these more complex ensemble models could provide accurate predictions for such cases.

Both ensemble models evaluated on the gold standard case dataset (n = 80) misclassified the same two cases (2.5%); one was mis-classified as FIP and another was mis-classified as non-FIP. We investigated these two cases further in order to understand why they may have been misclassified by the models. The history accompanying the ‘false positive’ case gave some indications that FIP was a realistic differential diagnosis. The clinical picture with this case was mixed: the cat presented with neurological signs (ataxia and confusion) and was a pedigree cat from a multi-cat household, thus a high FCoV titre was an expected finding. There were no signs of anaemia, however a moderately elevated AGP and hyperglobulinaemia indicated an inflammatory disease process. FIP, clinical toxoplasmosis or a bacterial aetiology were all suggested as differential diagnoses by the clinician based on the laboratory findings and clinical history. Records show that the clinical signs resolved and the cat survived at least five years post-testing for FIP; we conclude that this case was likely to be either clinical toxoplasmosis or a bacterial infection, both treatable with antibiotics. Importantly, we were able to conclude that this was not FIP as the case preceded the availability of the effective anti-viral treatments. The ‘false negative’ was a case where the clinical presentation was of neurological signs (acute ataxia) and uveitis (2 month onset) in a nine month old, male entire, domestic shorthair cat. Combined neurological and ocular FIP is an uncommon presentation of this disease. The primary clinician observed retinal changes in the left eye, marked brain abnormalities and inflammatory cells in a cerebrospinal fluid sample. Incidentally, this case was included in another project to develop a molecular diagnostic test for non-effusive FIP^14^, and also produced a false negative result in that study. FIP was confirmed in this case by histopathology on brain tissue at several sites; ocular tissue also exhibited changes consistent with FIP, though not conclusively. The laboratory diagnostic tests utilised, as well as the models developed in this study, aim to discriminate cases of what is termed ‘classic non-effusive FIP’. Therefore, the inability to detect a particularly atypical case of FIP, which does not produce the same systemic inflammatory responses as a classic disease manifestation, is unsurprising.

Non-effusive FIP is one of the most challenging conditions to diagnose for the companion animal practitioner. We demonstrate here that ML has great potential to effectively automate the interpretation of routine clinical laboratory data for diagnostic purposes and that the most robust models for this purpose are tree-based classification algorithms, specifically extreme gradient boosting (XGBoost) and random forests. The mixed ensemble model, comprising 100 base learners from each of the four algorithm types feeding into a random forest, performed marginally better than the XGBoost ensemble model which was evaluated at the validation stage. However, the difference in performance level was found to be extremely small, and both ensembles performed comparably when assessed against the two test datasets. Under closer scrutiny, the mixed ensemble takes account of more variables and variation in the dataset than the XGBoost models, which primarily prioritise FCoV titre and AGP concentration as the variables of major importance. In cases with more subtle signals in the remaining variables, which the XGBoost models are less likely to evaluate, the risk of misclassification increases. Our analysis of the ‘standard haematology and biochemistry’ dataset alone indicates that these nuanced patterns do indeed exist. Consequently, mixed ensemble models likely offer the greatest potential for future translation into clinical use. Additionally, the mixed ensemble is less computationally demanding in terms of production of the model. Both ensemble models displayed predictive performance far exceeding expectations, with model accuracy measures in the 90–100% bracket from inception. The panel of algorithms was selected in order to evaluate differences in predictive capabilities of each type of algorithm, with varying degrees of complexity. Models based on individual algorithms exhibit predictive power, however each algorithm has its strengths and weaknesses. Ensemble models generally perform better than any individual contributor model and tend to make superior predictions. The use of numerous models to produce an aggregated decision reduces the variance and bias that can be observed in a single model and so the present approach leads to more accurate predictions. The high levels of performance observed for all the models built further supports our proposal that this form of dataset is well suited to the application of ML approaches.

A major consideration when modelling this dataset was that the timepoints of disease progression at which the animals were sampled were unknown and these would be highly varied. Consequently, the clinical and laboratory data represented only a snapshot of the clinical picture at the time the animal presented to the primary clinician. In some cases, cats might have been sampled relatively early in the disease process, whereas in other cases the disease might have markedly progressed and the cat would have been near the end of its life. This necessarily contributes an additional, unquantifiable aspect to the variation in the dataset. In some cases of FIP, clinical signs are slow to develop and can be subtle, with the disease developing insidiously over a long period of time. While the majority of submissions represent well-progressed cases, some will be from cases in the early stages of disease. Unfortunately, early FIP cases may display an overlap in clinical signs and blood picture, with an even larger group of alternative diseases or disorders than late cases. As described, we excluded cases where specific anti-viral treatment was mentioned but we did not exclude cases receiving various palliative treatments. It was considered that some of these drugs could potentially have had an effect on the haematology and biochemistry markers, but no curative effect on the disease. Despite these complexities, we have shown that diagnostic models can be remarkably successful at discriminating FIP from non-FIP cases.

ML in medicine has come under scrutiny as a ‘black box’ approach to the generation of predictions and consequent clinical decision-making, as the internal working of models is not easily explained to those outside the ML field. In the cases of the algorithms that were implemented here, should it be required, it would be possible to interrogate the models and provide some understanding of how they arrive at a prediction for a specific case. Increasing our understanding of how the models derive their predictions and supporting the outcome from the model with both clinical and biological knowledge allows us to understand and explain the prediction in individual cases. It could be argued that this point is fundamental to our ability to use such models in a clinical setting. The interpretability of the models and the ability to accurately explain the models’ decisions is a critical factor that must be addressed if these systems are to be implemented in healthcare workflows.

## Conclusion

FIP is a complex clinical condition which remains a diagnostic challenge to practising veterinary surgeons. We have demonstrated that, in principle, ML can be effectively applied to the realm of FIP diagnostics. Insight has been shown as to which types of models may be best applied to this problem and which laboratory parameters may be particularly valuable. While further work is required before a deployable ML tool is available, encouragement can be taken from the high level of accuracy achieved by these preliminary models. In future, such models could be incorporated into computer or web-based applications and may be used as ‘expert systems’ for the benefit of veterinary clinicians engaged in the diagnostic process. Thus, ML offers the potential to form a useful additional layer to laboratory workflows and may in future be added to the arsenal of tools available for FIP diagnostics.

### Supplementary Information

Supplementary Information.

## Data Availability

The clinically sensitive data used in this study can be made available to academic institutions and other interested parties upon request. To obtain data, a formal request should be made to the corresponding author.

## Code availability

Code used in the study is available on GitHub at the following link https://github.com/ddunbar84/Dunbar_FIP_NonEffusive_ML, and can also be made available upon request.
