# Supplementary material for: Assessing the feasibility of applying machine learning to diagnosing non-effusive feline infectious peritonitis
Source: Sci Rep. 2024 Jan 30;14:2517. doi: 10.1038/s41598-024-52577-4 (PMC10827733; doi:10.1038/s41598-024-52577-4)
Supplement: Supplementary file 1 — Supplementary Information. [file 41598_2024_52577_MOESM1_ESM.docx]

## **Title**

Assessing the feasibility of applying machine learning to diagnosing non-effusive feline infectious peritonitis

## **Authors and Affiliations**

Dawn Dunbar ^a*^, Simon A. Babayan ^a^, Sarah Krumrie ^a^, Hayley Haining ^a^, Margaret J. Hosie ^b^ and William Weir ^a^

^a^ School of Biodiversity, One Health and Veterinary Medicine, College of Medical, Veterinary and Life Sciences, University of Glasgow, Glasgow, United Kingdom

^b^ MRC-University of Glasgow Centre for Virus Research, College of Medical, Veterinary and Life Sciences, University of Glasgow, Glasgow, United Kingdom

*Correspondence to dawn.dunbar@glasgow.ac.uk

## **Supplementary Information**

### Data Cleaning

The immunofluorescence assay (IFAT) to assess FCoV antibody titres is typically conducted over eight 2-fold dilutions from 1:10 to 1:1280. The assay is an indirect IFAT. Briefly, FCoV infected cells are fixed to glass slides, anti-FCoV cat serum is incubated on the slides (the Fab region binds to the FCoV expressed in the cells), non-bound serum is washed away and then attached serum is tagged at the Fc region with FITC labelled anti-cat polyclonal antibody, which is then read under UV light. Samples that still tested positive for antibody at the final dilution of 1:1280 had a titre of >1280 recorded in the LIMS. For analytical purposes, these were adjusted to an arbitrarily high titre of 1960, one half-dilution above the highest recorded dilution. This step was necessary, as the algorithms required numeric values to operate. A similar principle was applied to AGP measurements. The tests for AGP either capture the protein using a sandwich ELISA and measurements are calculated from a standard curve of optical densities or, previous to that, radial immunodiffusion was used, whereby the AGP is precipitated in an agar gel and the size of the precipitant ring is measured, with the concentration of AGP again being calculated from a standard line. The assay was swapped to an ELISA as the RID production was discontinued and the new method was fully validated before swapping over. Where values >3600 μg/ml were recorded (the maximum reading from the RID assay), a nominal value of 3601 μg/ml was assigned; this was also applied to ELISA readings >3600 μg/ml as the algorithms require numerical data. This change is unlikely to influence the outcome predictions, as the classic AGP level deemed significant for FIP diagnosis is typically >1500 μg/ml. Clinical pathology data were recorded in the form of continuous numerical variables and did not require additional steps to prepare the data.

### *Exploratory Data Analysis*

Principal Component Analysis (PCA), an unsupervised clustering method, was employed to reveal broad patterns in the dataset. Scatterplots were generated for each variable combination in the complete dataset prior to removal of any features to evaluate correlation and covariance (data not provided). A correlation plot was generated displaying the interactions between all variables included in the final models (see Supplementary Fig. S1). Stratified density plots were generated to display the distribution of data within each response variable and data partition (see Supplementary Fig. S2).

### Data Pre-processing for Modelling

Data points for each parameter were centred and scaled prior to use in the algorithms, in order to make different test results comparable, thereby facilitating analysis on a single data modelling framework. For example, feline coronavirus antibodies were measured using a primary dilution of 1:10, then 2-fold dilutions up to 1:1280. This test-range was, therefore, entirely different to other parameters, such as albumin to globulin ratio, which typically scaled between 0.2 and 1, with rare outliers in extreme clinical conditions. Mean-centred scaling was used to reduce the variation in the data to between -1 and 1, with 0 being the mean of the standardised data. It is important to appreciate that this pre-processing step is reversible, so that the original data can be retrieved when interrogating the models.

**Supplementary Table S1 - Machine Learning Algorithms and tuning parameters.** Details of name and type of algorithm and the hyperparameter tuning grid values, dependant package, package version and Caret model function of algorithms used in the trained base models and meta-models evaluated in this study.

|  | Algorithm name | Algorithm type | Tuning hyperparameter | Hyperparameter setting | Dependant Package | Version | Caret Model function |
| --- | --- | --- | --- | --- | --- | --- | --- |
| Base Models | Logistic regression | Regression |  |  | “stats” ^1^ | 4.1.2 | “glm” |
|  | Naïve Bayes | Probabilistic | laplace  usekernel  adjust | 0  True, False  0, 0.25, 0.5, 0.75, 1 | “naivebayes”^2^ | 0.9.7 | “naivebayes” |
|  | Support Vector Machine | Discriminative classifier | Cost | 0.25, 0.5 | “e1071”^3^ | 1.7.9 | “svmLinear2” |
|  | Random Forest | Tree-based | mtry | 1:11 | “randomForest” ^4^ | 4.6.14 | “rF” |
|  | Xtreme Gradient Boosting | Ensemble Tree-based | nrounds  eta  max_depth  gamma  colsample_bytree  min_child_weight  subsample | 200, 300, 400, 500, 600, 700, 800, 900, 1000  0.05  3,4,5  0  1  1  0.5,0.75 | “xgboost”^5^ | 1.0.5.2 | “xgbTree” |
| Meta-models | Random Forest | Tree-based | mtry | 40:50 | “randomForest” ^4^ | 4.6.14 | “rF” |

**Supplementary Figure S1– Package List -** List of all packages loaded and utilised in the production of the models and visualisations within this paper and the version number as used in modelling.

Additional attached packages:

furrr v. 0.2.3

future v. 1.23.0

doParallel v. 1.0.14

iterators v. 1.0.13

foreach v. 1.5.1

ggbiplot v. 0.55

scales v. 1.1.1

devtools v. 2.4.3

usethis v. 2.1.5

ggfortify v. 0.4.14

ggthemes v. 4.2.4

ggridges v. 0.5.3

gridExtra v. 2.3

ggpubr v. 0.4.0

Rmisc v. 1.5

plyr v. 1.8.6

randomForest v. 4.6-14

xgboost v. 1.5.0.2

kernlab v. 0.9-29

e1071 v. 1.7-9

gbm v. 2.1.8

pROC v. 1.18.0

stringi v. 1.7.6

caretEnsemble v. 2.0.1

caret v. 6.0-90

lattice v. 0.20-45

magrittr v. 2.0.1

dataMaid v. 1.4.1

knitr v. 1.37

forcats v. 0.5.1

stringr v. 1.4.0

dplyr v. 1.0.7

purrr v. 0.3.4

readr v. 2.1.1

tidyr v. 1.1.4

tibble v. 3.1.6

ggplot2 v. 3.3.5

tidyverse v. 1.3.1

Base R packages:

parallel v. 4.1.2

grid v. 4.1.2

stats v. 4.1.2

graphics v. 4.1.2

grDevices v. 4.1.2

utils v. 4.1.2

datasets v. 4.1.2

methods v. 4.1.2

base v. 4.1.2

**Supplementary Table S2 – Variables removed from feature list and reason for removal.** Details of variable name, description of variable and reason for exclusion from model building.

| Variable | Description | Reason for removal |
| --- | --- | --- |
| RBC | Red blood cell count | Highly correlated with haemoglobin and more susceptible to artefactual change. |
| HCT | Haematocrit | Highly correlated with haemoglobin and more susceptible to artefactual change. |
| MCV | Mean corpuscular volume | Highly correlated with haemoglobin and more susceptible to artefactual change. |
| MCH | Mean cell haemoglobin | Highly correlated with haemoglobin and more susceptible to artefactual change. |
| MCHC | Mean cell haemoglobin concentration | Highly correlated with haemoglobin and more susceptible to artefactual change. |
| WBC | Total white blood cell count | Correlated with differential cell counts but less informative so differential counts used instead. |
| Band neutrophils | Band neutrophil count | Less than 25% of cases measured/recorded a band neutrophil count. Measure would not provide useful information to the models. |
| Basophils | Basophil count | Less than 20% of cases measured/recorded a basophil count. Measure would not provide useful information to the models. |
| Normoblasts | Normoblast count | Less than 10% of cases measured/recorded a normoblast count. Measure would not provide useful information to the models. |
| Total protein | Total protein concentration | Incorporates albumin; albumin and A:G ratio used instead as more informative. |
| Globulin | Globulin concentration | Albumin and A:G ratio proved more informative; globulin measure rendered redundant as represented by A:G ratio. |
| Reason | Clinical history / suspected diagnosis provided on submission form by submitting clinician. | This will be used in future work to try to extract clinical signs. Cases do not consistently have this information submitted or recorded. |


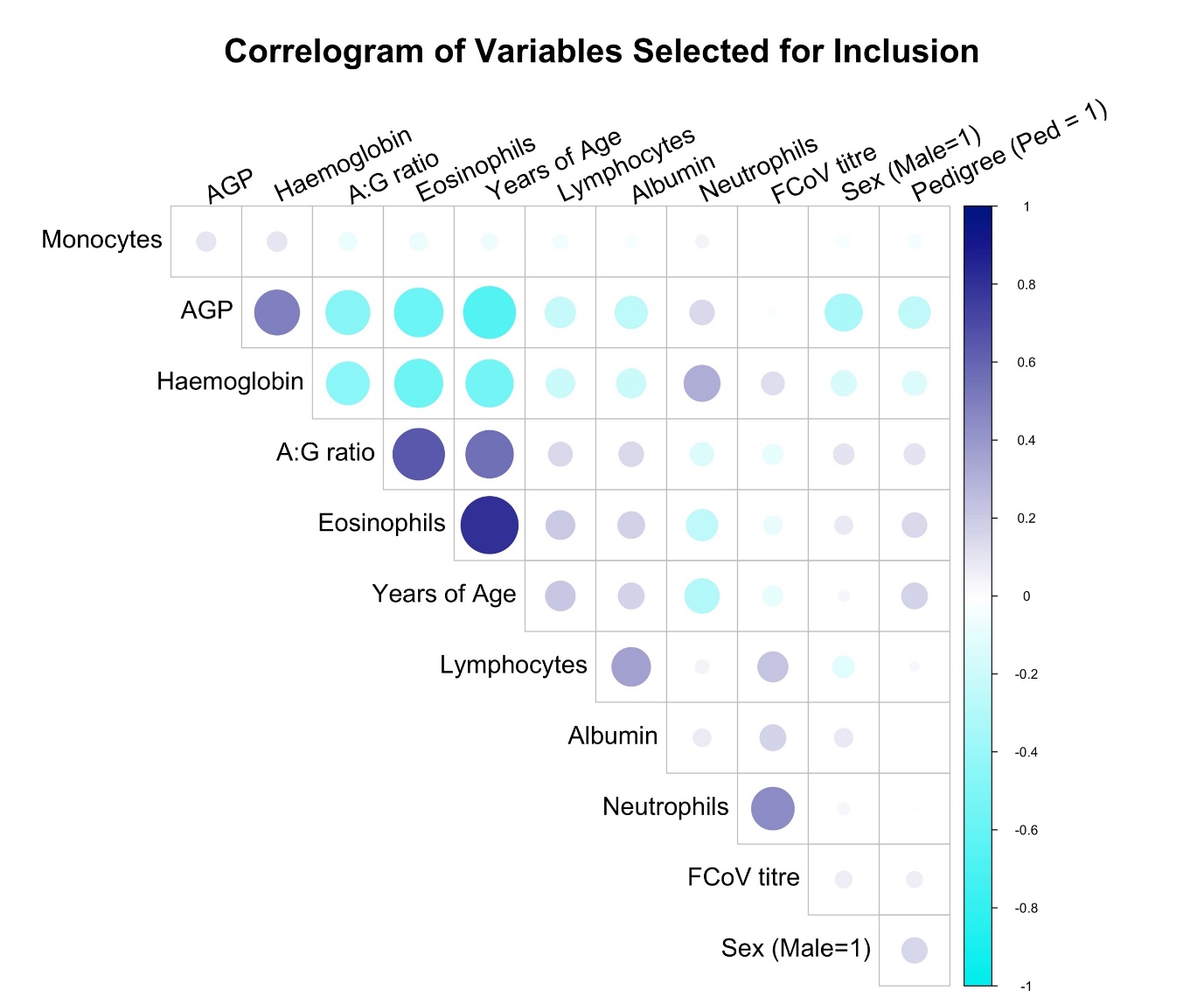
**Supplementary Figure S2 – Correlation plot.** Correlation intensity of variables, dark blue illustrates a strong positive correlation, light turquoise illustrates a strong negative correlation.

**Supplementary Table S3 – McNemar test results for model comparisons.**

|  | Validation Dataset | | | Expert Opinion Dataset | | | Gold Standard Dataset | | |
| --- | --- | --- | --- | --- | --- | --- | --- | --- | --- |
| Model comparison | McNemar Chi-squared statistic | Degrees of freedom | McNemar  p-value | McNemar Chi-squared statistic | Degrees of freedom | McNemar  p-value | McNemar Chi-squared statistic | Degrees of freedom | McNemar  p-value |
| Mixed Ensemble vs All variable LR | 0.868 | 1 | 0.352 | 0.232 | 1 | 0.630 | 0 | 1 | 1 |
| Mixed Ensemble vs FCoV LR | 0.085 | 1 | 0.771 | 0.413 | 1 | 0.521 | 0.5 | 1 | 0.48 |
| XGBoost Ensemble vs All variable LR | 1.211 | 1 | 0.271 | 0.418 | 1 | 0.518 | 0 | 1 | 1 |
| XGBoost Ensemble vs FCoV LR | 0.216 | 1 | 0.643 | 0.654 | 1 | 0.419 | 0.5 | 1 | 0.48 |
| Mixed Ensemble vs XGBoost Ensemble | 1.333 | 1 | 0.248 | 0.5 | 1 | 0.48 | -* | -* | -* |
| Mixed Ensemble (all Variables) vs Mixed Ensemble (no FCoV, no AGP) | 10.105 | 1 | 0.0015 | 12.033 | 1 | 0.0005 | 0.25 | 1 | 0.617 |
| XGBoost Ensemble (all Variables) vs Mixed Ensemble (no FCoV, no AGP) | 9.121 | 1 | 0.003 | 12.033 | 1 | 0.0005 | 0.25 | 1 | 0.617 |

| Evaluation Dataset | Model | Base Learner Training Resample Accuracy (%) (Range) | Validation  Accuracy (%)  (95% CI) | Accuracy (%) (95% CI) | Sensitivity (%) (95% CI) | Specificity (%) (95% CI) | Cohens Kappa (κ) | AUC  (95% CI) |
| --- | --- | --- | --- | --- | --- | --- | --- | --- |
| Training & Validation | Mixed Ensemble – minus FCoV & AGP | 99.06  (97.98-99.81) | 92.91  (90.87-94.62) | - | 94.74  (91-97) | 91.96  (89-94) | 84.64 | 0.934  (0.916-0.951) |
|  | LR1 – all predictors | - | 98.58  (97.48-99.29)* | - | 97.37  (95-99) | 99.22  (98-100) | 96.85 | 0.983  (0.973-0.993) |
|  | LR2 – FCoV Antibodies | - | 98.20  (96.99-99.01)* | - | 98.87  (97-100) | 97.84  (96-99) | 96.02 | 0.984  (0.975-0.993) |
| Expert Opinion Test Set | Mixed Ensemble – minus FCoV & AGP | - | - | 92.27  (89.15-94.72) | 96.55  (92-99) | 89.71  (85-93) | 93.93 | 0.931  (0.907-0.956) |
|  | LR1 – all predictors | - | - | 98.45  (96.66-99.43)* | 97.24  (93-99) | 99.18  (97-100) | 96.69 | 0.982  (0.976-0.996) |
|  | LR2 – FCoV Antibodies | - | - | 98.97  (97.38-99.72)* | 98.62  (97-100) | 99.18  (95-99) | 97.80 | 0.990  (0.978-1.0) |
| Gold Standard Cases | Mixed Ensemble – minus FCoV & AGP | - | - | 92.5  (84.39-97.2) | 90.91  (71-99) | 93.10  (83-98) | 81.71 | 0.920  (0.850-0.990) |
|  | LR1 – all predictors | - | - | 95.00  (87.69-98.62)* | 95.45  (77-100) | 94.83  (86-99) | 87.80 | 0.951  (0.898-1.000) |
|  | LR2 – FCoV Antibodies | - | - | 97.50  (91.26-99.7)* | 100  (85-100) | 96.55  (88-100) | 93.90 | 0.983  (0.959-1.000) |

**Supplementary Table S4 - Model accuracies for the mixed ensemble without FCoV and AGP, and both Logistic regression models with each dataset.** Accuracy, sensitivity, specificity, kappa and AUC of each model are detailed.

**References**

1 R: A language and environment for statistical computing (R Foundation for Statistical Computing, Vienna, Austria, 2019).

2 Majka, M. *naivebayes: High Performance Implementation of the Naive Bayes Algorithm in R*, <https://CRAN.R-project.org/package=naivebayes> (2019).

3 David Meyer, E. D., Kurt Hornik, Andreas Weingessel, Friedrich Leisch. *e1071: Misc Functions of the Department of Statistics, Probability Theory Group (Formerly: E1071), TU Wien*, <https://CRAN.R-project.org/package=e1071> (2021).

4 Andy Liaw, M. W. Classification and Regression by randomForest. **2**, 18-22. https://CRAN.R-project.org/doc/Rnews/ (2002).

5 Chen, T. & Guestrin, C. in *Proceedings of the 22nd ACM SIGKDD International Conference on Knowledge, Discovery and Data Mining.* 785-794 (ACM).
